# Self-reported emotion regulation difficulties are associated with mood but not with the biological stress response to thin ideal exposure

**DOI:** 10.1371/journal.pone.0199769

**Published:** 2018-06-27

**Authors:** Nadine Humbel, Nadine Messerli-Bürgy, Kathrin Schuck, Andrea Wyssen, David Garcia-Burgos, Esther Biedert, Julia Lennertz, Andrea H. Meyer, Katherina Whinyates, Bettina Isenschmid, Gabriella Milos, Stephan Trier, Dirk Adolph, Jan Cwik, Jürgen Margraf, Hans-Jörg Assion, Tobias Teismann, Bianca Ueberberg, Georg Juckel, Judith Müller, Benedikt Klauke, Silvia Schneider, Simone Munsch

**Affiliations:** 1 Clinical Psychology and Psychotherapy, Department of Clinical Psychology, University of Fribourg, Fribourg, Switzerland; 2 Faculty of Psychology, Mental Health Research and Treatment Center, Ruhr-Universität Bochum, Bochum, Germany; 3 Department of Psychology, Division of Clinical Psychology and Epidemiology, University of Basel, Basel, Switzerland; 4 Privatklinik Schützen, Rheinfelden, Switzerland; 5 Kompetenzzentrum für Essstörungen und Adipositas (KEA), Spital Zofingen, Zofingen, Switzerland; 6 Klinik für Psychiatrie und Psychotherapie, Universitätsspital Zürich, Zürich, Switzerland; 7 Privatklinik Aadorf, Aadorf, Switzerland; 8 LWL-Klinik Dortmund, Department of Psychiatry, Psychotherapy and Psychosomatic Medicine, Dortmund, Germany; 9 Department of Psychiatry, Psychotherapy and Preventive Medicine, Ruhr University Bochum, LWL University Hospital, Bochum, Germany; 10 Christoph-Dornier-Clinic for Psychotherapy, Münster, Germany; Universitat Wien, AUSTRIA

## Abstract

**Background:**

Difficulties in emotion regulation have been related to psychological and physiological stress responses such as lower mood and lower parasympathetic activation (HF-HRV) under resting condition, but evidence on the potential link to the hypothalamic-pituitary-adrenal (HPA) axis functioning and to physiological stress responses during a stress task is still scarce. The aim of the study was to investigate stress responses in young women when confronted to a daily stressor such as exposure to thin ideals and to understand the role of correlates of self-reported trait-like emotion regulation difficulties (ERD).

**Methods:**

Heart rate variability (HRV) and salivary cortisol data were collected in a sample of 273 young women aged 18–35 with and without mental disorders during a vivid imagination of thin ideals (experimental condition) or landscapes (control condition). Changes in mood states were measured on a visual analogue scale (0–100). Correlates of trait-like ERD were self-reported using the Difficulties in Emotion Regulation Scale (DERS).

**Results:**

Participants with higher ERD showed a stronger decline in self-reported mood after vivid imagination of thin ideals compared to participants with lower ERD in the experimental condition but also a stronger increase of positive mood with increasing ERD in the control condition. ERD were not related to baseline HF-HRV or baseline salivary cortisol levels nor to any physiological response during and after the imagination of thin ideals.

**Discussion and conclusion:**

The results corroborate the role of ERD regarding the immediate psychological impact of daily stressors. Exposition to daily stressors in the laboratory results in discrepant psychological and physiological reactivity. Future studies should investigate under what conditions the complex interrelations between immediate and long-term ERD and biological activation are amenable to assessment in a laboratory setting. The additive effects of multiple exposition to stressors, such as thin ideals in daily life, also need to be addressed.

## Introduction

Stress reactivity represents an important mechanism to cope with environmental challenges and threats. Faced with a challenging situation, both physiological and psychological processes are triggered to different degrees and interact in order to enable the individual to cope with acute and chronic stress [1].

With respect to psychological mechanisms influencing stress reactivity, emotion regulation plays an important role. Gross [2] defined emotion regulation as encompassing “*the processes by which individuals influence which emotions they have*, *when they have them*, *and how they experience and express these emotions*” (pp. 275). Adaptive emotion regulation has been summarized in a multidimensional model by Gratz and Roemer [3] and emphasizes four dimensions: the awareness and understanding of emotional states, the acceptance of emotions, the ability to inhibit impulsive behavior and to maintain goal-directed behavior, and the flexible use of adaptive and appropriate strategies to modulate emotional responses. Impairments in any of these dimensions stand for a rigid control, for suppression of emotions or for an inadequate use of strategies reflecting trait-like emotion regulation difficulties (ERD). ERD are widely accepted as a transdiagnostic core feature involved in the onset and maintenance of psychopathology [4–6]. Prior research showed evidence that ERD are related to a broad range of mental disorders [7] such as depressive disorders and anxiety disorders [4,8] or eating disorders such as anorexia nervosa (AN) and bulimia nervosa (BN) [9,10]. In these studies, ERD were measured by well-validated self-report questionnaires to assess different facets of emotion regulation and its dysfunction such as the Difficulties in Emotion Regulation Scale (DERS) [3].

Lately, there has been increasing interest in the interaction between ERD and biological mechanisms when faced with situational challenges. The capacity of the organism to adequately react to the environment depends on the flexible neural network system called autonomic nervous system (ANS) which is connected to the amygdala and the medial prefrontal cortex and is indexed by heart rate variability (HRV) [11,12]. Based on Thayer and Lane’s *Model of Neurovisceral Integration* [13], high frequency heart rate variability (HF-HRV) represents parasympathetic activation (PNS) and has been identified as a marker of emotion regulation [13]. In other words, higher HF-HRV in resting state and greater HF-HRV changes when facing situational challenges are related to emotion regulation attempts and stand for a more adaptive response and subsequent recovery from a stressor [11]. In contrast, lower HF-HRV, during resting conditions, reflects impairments of the regulatory role of the ANS. Increased ERD are associated with limited inhibition capacity of the PNS [1,14], in healthy individuals [15] and in mental disorders such as eating disorders [16]. In contrast, data on HRV change is limited and little is known about the link between ERD and HRV change [16]. Those studies investigating the relation between ERD and ANS responses relied on standardized stress tasks [17], while only few applied ecologically valid paradigms eliciting daily moderate stress conditions by personal recall or picture viewing methods [18]. Berna, Ott and Nandrino [19] investigated HF-HRV changes during an emotion-elicitation video clip-based stress task in a sample of 63 healthy undergraduate students at the age of 18 to 27. Prior to the exposition to three consecutively presented anger-inducing short video clips (FilmStim database), Berna and colleagues [19] assessed ERD and divided participants into lower and upper quartiles depicting well-developed and limited emotion regulation capacities respectively according to the DERS. They further measured skin conductance and HF-HRV. The result was that, if controlled for the influence of depression, anxiety and age, the group in the upper quartile of ERD showed less change in HF-HRV between an elicitation task and recovery than the low ERD group and thus took longer to recover after emotion elicitation. In contrast to prior research, there was no difference between groups at rest. According to Berna and colleagues [19], enduring ERD causes prolonged HRV recovery and thus a decrease of resting HRV level over time due to an extended overload of the stress system.

Up to now, data on the association between ERD and physiological stress responses focused on the assessment of HRV as a correlate of the autonomic activation and neglected the stress responses of the hypothalamic-pituitary-adrenal (HPA) axis indexed by the increased release of salivary cortisol in response to a challenge [20]. The HPA axis is especially important in terms of interactions of ERD and biological stress response [21], as the HPA system is influenced by stress and by higher order cognitive processes such as the evaluation of the perceived information [22]. We found only one study by Lam and colleagues [23] of healthy male and female students, where higher scores of maladaptive trait emotion regulation strategies predicted heightened cortisol release after a social speech task.

Data on the interrelation between ERD and biological mechanisms of individuals facing standardized stress situations is very partial and knowledge on the processes during moderately stressful daily events remains scarce. Apart from emotion eliciting video or picture viewing [19,24], engagement in aversive or in worrisome mental imagery has been shown to be an important daily stressor [25]. Therefore, this laboratory study seeks out to examine the effect of exposure to thin ideals in media and subsequent vivid imagery of thin ideals in young females with different levels of ERD. Based on prior research regarding the stress inducing effect of body image exposure [26,27], we expected exposure to thin ideals in media to be a moderate daily stressor, fulfilling criteria of ego-involvement in young females in the age of risk between 18 to 35 years [28–31]. To intensify the effect of exposure, we instructed participants to a subsequent vivid imagination of the most attractive female bodies previously seen in the magazines [32]. Our assumption was confirmed in our previous study where combined exposition and vivid imagery of thin ideals led to self-reported stress responses in terms of impaired mood, body satisfaction and body-related cognitive distortions in healthy women [33]. Therefore, in the current study, we considered vivid imagination of thin ideals to be a moderate daily stressor triggering stress responses in our female sample and favored ecological validity over a fully standardized exposure. In order to control for effects of quality of exposure, we additionally asked for self-report of the vividness of imagery and carried out an implicit memory test on the material seen during the exposure.

In this framework, the aim of the present study is fourfold: *First*, we investigated whether self-reported stress response in terms of mood is related to increased trait-like ERD measured by DERS. *Second*, we examined, whether increased ERD are associated with impaired PNS functioning. *Third*, we aimed at extending knowledge on the relation between ERD and the dysregulation of the HPA axis [34,35]. We included measures of HRV as well as of salivary cortisol in order to detect activation of both branches of the stress response system. *Fourth*, we investigated the association between ERD and psychological and biological stress responses to moderately stressful daily stressors in a large sample. This consisted of healthy and young women suffering from a mental disorder (eating disorders such as AN and BN, depressive or anxiety disorder) thereby potentially reflecting a wide range of ERD. The interest of the current study is therefore based on the impact of ERD and not on the categories of mental disorder.

Specifically, we hypothesized that participants with high levels of self-reported ERD are characterized by a more pronounced decrease of mood and impaired PNS and HPA functioning at both rest and during stress. This should be observed in lower resting HRV level and by smaller HRV changes from baseline to vivid imagination and from vivid imagination to recovery. We should also see higher salivary cortisol levels at rest and smaller salivary cortisol differences between the periods before (baseline) to after the vivid imagination (recovery) than participants with low ERD compared to a neutral condition, where no stress response independent of ERD is expected.

As advised in previous relevant studies [36,37], we controlled for the effects of age, BMI, use of hormonal contraceptives and medication with effects on the central nervous system (pharmaceuticals and psychopharmaceuticals) on biological parameters. We further considered the effect of the vividness of imagery and the implicit recognition of pictures presented during the exposure in order to approximate the strength of stress induction [33,38].

## Methods

### Participants

275 young women between the age of 18 and 35 (*M* = 22.87, *SD* = 3.94) were recruited over a period of 3 years in Switzerland and Germany. Altogether 174 female inpatients and outpatients were recruited in four Swiss clinics in the cantons of Zurich, Aargau and Thurgau. In Germany six clinical institutions in the Ruhr area collaborated and all incoming patients were routinely asked to participate. The clinical sample consists of 112 patients with eating disorders (AN *n* = 58 and BN *n* = 54) and 62 patients with mixed non eating disorders (depressive disorders *n* = 38, anxiety disorders *n* = 21, somatoform disorders *n* = 3). The sample also includes 101 healthy female students recruited in Switzerland at the University of Fribourg and from several vocational schools in the Canton of Fribourg, Switzerland. This study was approved by the Ethics Research Committee of the Departement of Psychology at the University of Fribourg (reference no. 2012_001), the Ethics Committees of the Cantons of Fribourg (reference no. 023/12-CER-FR), Zurich (reference no. 2013–0457), Aargau (reference no. 2013/057) and Thurgau (reference no. 2013/24) and the Ethics Committee of the Faculty of Psychology at the Ruhr-University Bochum in Germany (reference no. 142). The study followed the guidelines of the Declaration of Helsinki and the Good Clinical Practice Directive of Switzerland. Participation was voluntary and each participant signed the informed written consent. The trial is registered in the German Clinical Trials Registry (trial number: DRKS00005709).

362 patients were informed about the study and approximately 1000 healthy participants where reached through flyers. After applying the exclusion criteria, namely age (under 18 or over 35 years), insufficient language skills, current pregnancy or breastfeeding, psychotic disorder, serious somatic illness having an effect on eating, mood or biological measures such as cancer or hyperthyroidism, past bariatric surgery or pharmacological treatment of arrhythmias, we were left with 213 patients and 128 healthy subjects. A total of 39 patients and 27 healthy subjects had to be excluded during the course of the study because of withdrawal of participation (*n* = 19) or since they met one or more exclusion criteria (*n* = 47). Participants of the mixed clinical group (depressive disorders, anxiety disorders or somatoform disorders) were excluded if they currently fulfil the criteria of an eating disorder. Eating disorder patients were included only if they met the full criteria of AN or BN. Exclusion criteria for healthy participants were the presence of any current mental disorder or past eating disorder assessed by the DIPS (Diagnostisches Interview für psychische Störungen) [39] or a body dysmorphic disorder assessed by the SCID (Structured Clinical Interview for DSM-IV Axis I, Section G, Body Dysmorphic Disorder) [40], or a total score higher than 2.5 at the EDE-Q (Eating Disorder Examination Questionnaire) [41] according to Fischer et al. [42]. All participants received a financial compensation (patients) or credit points (undergraduate students) and were subsequently debriefed about the purpose of the study.

### Procedure

The study is part of the multi-site cross and longitudinal experimental trial “Psychological and physiological consequences of exposure to mass media in young women–the role of moderators”. A full description of the overall study sample, protocol, all measurements and procedure is provided in Munsch [32].

**Fig 1:**
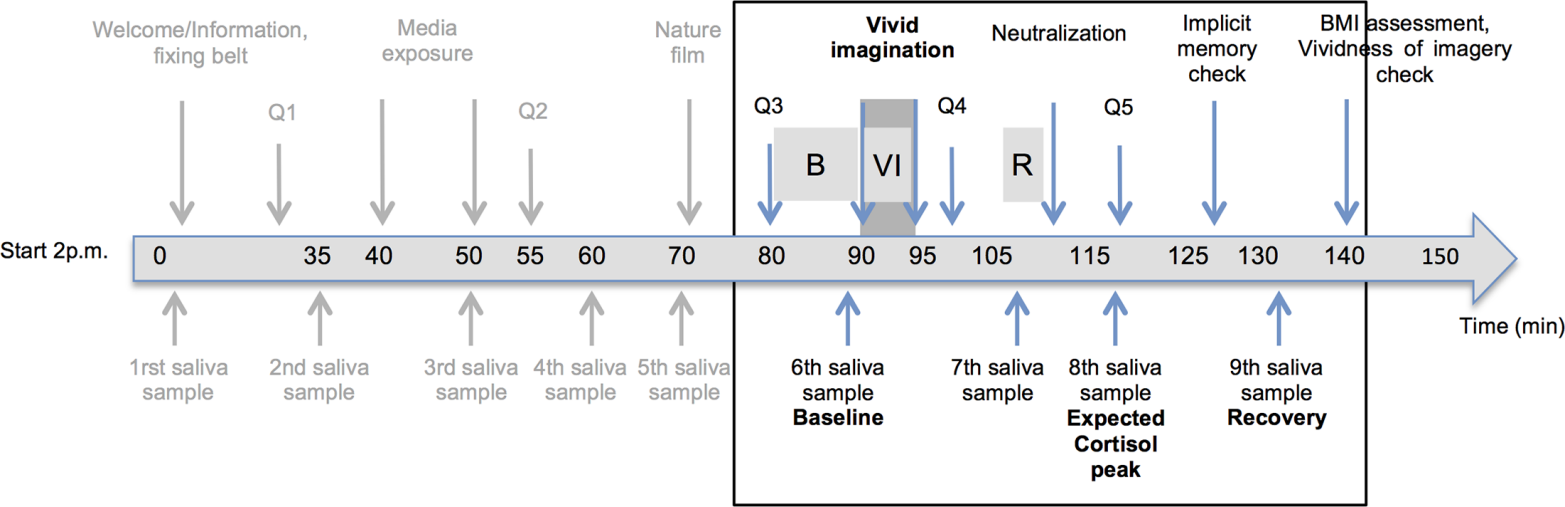
Experimental procedure with exact saliva sampling time points. Q = Questionnaires 1 to 5 including mood questionnaire; HRV segmentation: B = Baseline, VI = Vivid imagination, R = Recovery. The part of the experimental procedure used for this substudy is highlighted.

In all study units, the protocol was administered as follows: After signed informed consent, all participants were randomly assigned to a thin ideal exposure condition (EC: exposure to a fashion magazine followed by vivid imagination of the thin ideals) or neutral exposure condition (CC: exposure to a nature magazine followed by vivid imagination of landscapes). Participants received written information about the procedure and were told that the study examines mental well-being and psychophysiological stress reactivity relating to body image satisfaction in young adults. They were only informed about the objectives of the study and the experimental manipulation after participation. Participation included a diagnostic interview, the completion of a set of online questionnaires and an experimental testing day. Diagnostic interviews were conducted either by telephone or face-to-face. Participants were interviewed shortly after agreeing to participate and within the first weeks of beginning treatment for the patient group. One week after the interview, self-report questionnaires, such as DERS [3], were sent to all participants using an open source online survey tool. Healthy participants were invited to an individual testing afternoon the following week at the Laboratory of the University of Fribourg and patients to an individual testing at the corresponding clinical institution. Participants received oral and written information on physiological assessment prior to the experimental procedure. This included the following restrictions: participants were instructed not to drink anything else but water, eat, brush their teeth, use mouth-rinse or smoke in the hour preceding the experimental procedure. The experimental procedure took place between 2 and 4.30 p.m. with respect to circadian rhythm of the HPA axis and the ANS system. Time stamps of each step of the experimental procedure were computer-based. Fig 1 highlights the parts of the experimental procedure after exposure to the thin ideals used for the purpose of this substudy.

Physiological measurements and repeated administration of self-report questionnaires on body image, eating behavior and mood (Brief Mood Scale) [43] were assessed repeatedly before and after media exposure and subsequent vivid imagination. Participants were asked to put on the Electrocardiogram (ECG) breast belt for heart rate (HR) measurement at 10 minutes after the beginning of the experimental procedure and were told not to touch or refasten the belt once adjusted. Heart rate was recorded continuously during the whole experimental procedure. Nine saliva samples for salivary cortisol were collected in order to capture peak and recovery phases over exposure periods [44], and three measurements were included in this study (Fig 1). For each saliva sampling, participants were told to keep the cotton roll in the mouth for 2 minutes, avoiding any contact with other body parts.

Media exposure with fashion (EC) respectively nature magazines (CC) started 40 minutes after the beginning of the experiment. Media exposure was inspired by the waiting room study of Turner et al. [30]. Participants were asked to wait for 10 minutes in a second room, being told that baseline HR measures were assessed during this period, and were explicitly told to look at the pictures in the magazine they were given. During recovery time between magazine viewing and vivid imagination an 8 minute nature movie was presented. Vivid imagination took place 40 minutes after media exposure. The vivid imagination procedure was conducted in accordance with Radomsky et al. [45], Shafran et al. [46] and Coelho et al. [47] aiming to induce the cognitive distortion ‘Thought-Shape Fusion’ (TSF). Participants were given standardized instructions related to either the thin ideal exposure condition during vivid imagination (Vogue Germany) or neutral exposure condition during this time period (Geo Special Germany). Participants were asked to close their eyes and were instructed to remember and vividly imagine for 4 minutes the most attractive thin ideals (EC) or impressive landscapes (CC) referring to the magazine previously viewed. Instructions were given to deepen the detailed imagination. Following vivid imagination, participants were asked to write down a short description of the thin ideals or the landscape they just imagined. They were told to start the description with “*I imagine…*”. Shortly after vivid imagination, participants were given the opportunity to neutralize their feelings during 5 minutes and were provided with examples of neutralization activities such as exercising, drawing pictures, body checking and mental neutralization. Participants then completed a picture recognition task to check whether they viewed the images presented during the exposure. At the end of the experimental procedure, weight and height were measured for BMI assessment and participants were asked to rate the vividness of the imagination of the pictures viewed during exposition.

### Instruments

#### Diagnostic interview

The DIPS [39] is a structured interview based on the Diagnostic and Statistical Manual of Mental Disorders (DSM-IV-TR) [48] with an interrater reliability ranging from .57 to .92 and retest-reliability ranges from Cohen’s Kappa .35 to .94 [39]. For the purpose of our study, the DIPS EDs section was adapted according to the Diagnostic and Statistical Manual of Mental Disorders 5 (DSM-5, unpublished section, available by the authors) [49], whereas DIPS and thus criteria according to DSM-IV-TR was applied for all other mental disorders. Interviewers were trained and supervised by the principal investigators, and 10% of the interviews were coded independently twice by two raters based on the audio recordings of the interviews. Interrater reliability for primary diagnoses was high and amounted up to Fleiss K = .850, while interrater reliability including primary and comorbid diagnoses was satisfactory with Fleiss K = .803. Values were calculated based on independent ratings and the interviewer’s ratings.

#### Self-reported trait-like emotion regulation difficulties

Participants completed the German translation of the DERS [3,50] one week prior to the experimental procedure. The 36-items questionnaire is a reliable instrument for the assessment of self-reported emotion regulation and its dysfunction. Answers are given on a 5-point scale ranging from “almost never” (1) to “almost always” (5). 11 items needed reverse coding. The DERS scores range from 36 to 180, higher total scores suggesting greater problems with emotion regulation. The items load on 6 factors: the subscales ‘Goals’ (*When I’m upset*, *I have difficulty getting work done*), ‘Impulse’ (*When I'm upset*, *I feel out of control*), Awareness (*I pay attention to how I feel*), ‘Clarity’ (*I have difficulty making sense out of my feelings*), ‘Non-acceptance’ (*When I’m upset*, *I become angry with myself for feeling that way*) and ‘Strategies’ (*When I'm upset*, *I believe there is nothing I can do to make myself feel better*). The DERS shows high internal consistency in a healthy sample (ɑ = .86) and good test-retest reliability of .74 [3]. Internal consistency in our sample was satisfying with Cronbach Alpha = .97.

#### Self-reported mood

The Brief Mood Scale, a modified version of the Three Dimensions Affect scale [43], was used for momentary assessment of mood. Two dimensions of mood, valence and calmness, were measured on a bipolar visual analogue scale (0–100). A total score was computed from the mean of the two dimensions, higher scores indicating higher positive mood. Two measurement time points Q3 and Q4 were used (see Fig 1). Cronbach’s alpha was .94 and .95, respectively.

### Physiological measures

#### Heart rate variability

ECG was continuously recorded using movisens ekgMove sensors (ambulatory monitoring system; movisens GmbH, Karlsruhe, Germany) with a sampling frequency of 1024Hz. The sensors were attached to the chest using a flexible belt with dry electrodes. All sensor data were saved and stored using the Unisens data format [51], which is a universal and generic format suitable for recording and archiving sensor data from various recording systems. The continuous ECG data were visually inspected and divided into different segments using the UnisensViewer software (http://www.unisens.org/index.php) and a Matlab-related toolbox (unisens4matlab; Matlab R2015b). In order to perform the segmentation, timestamps were recorded during the experimental manipulation. Segments of interest included a 10 minutes baseline measurement before vivid imagination, a 4 minutes recording during vivid imagination and a recovery period of 2 minutes following vivid imagination (Fig 1). HRV was extracted from interbeat interval data using the Kubios HRV 2.2 software (University of Eastern Finland, Kuopio, Finland). Inter-beat (R-R) time series were detrended using a smooth priors detrending method (λ = 500) and manually corrected for artifacts. Spectral values including high frequency band (HFHR (ms2) 0.15–0.40 Hz) and low frequency band (LFHR (ms2) (0.04–0.15 Hz)), total power (TPHR), and LF/HF ratio of HRV were then calculated by using the autoregressive method.

#### Salivary cortisol

Salivary Cortisol was collected using Salivette sampling devices (Sarstedt, Nümbrecht, Germany). Participants received oral and written instructions on how to handle the salivettes prior to each sample. Each cotton roll was chewed during exactly 2 minutes. The samples were taken at -2 (baseline), +20 (expected peak) and +35 minutes (recovery). Time points were recorded by hand as well as by computer. Salivary cortisol samples were stored at the respective locations, and regularly sent for analysis to the Laboratory of University of Zürich (Swiss samples) and to the Laboratory of the Department of Genetic Psychology at Ruhr-University Bochum (German samples). Samples were frozen at –20°C prior to laboratory testing. Biochemical analyses were conducted using Cortisol Luminescence Immunoassay. Lower level of detection was .276 nmol/L. Intraassay and interassay coefficients of variation were both < 5% for cortisol, sensitivity was at 0.004 μ g/dl.

### Covariates

#### Body mass index (BMI)

Weight and height of all participants were assessed using an electronic personal scale (Seca 899, Basel, Switzerland) and a stadiometer (Seca, Basel, Switzerland). Participants were asked to take their shoes off for these measurements. BMI was then calculated as body mass index by weight in kilograms divided by the square of the body height in meters (kg/m^2^).

#### Use of hormonal contraceptives

The use of hormonal contraceptives was assessed at the beginning of the experiment. Participants were asked if they use any hormonal contraceptive including oral contraceptives, subcutaneous contraceptives, intravaginal and intrauterine contraceptives. A dichotomous variable was built to identify whether any hormonal contraceptive was used. 169 participants (61.9%) were under hormonal contraceptive.

#### Medication

Participants were asked about their medication intake one week prior to the experiment by online questionnaire. The exact name and purpose of the medication was assessed and a new variable was built assessing whether or not medication with a potential effect on the cardiovascular system and/or HPA axis was currently consumed. Medication with a potential effect on the cardiovascular system and/or HPA axis, including triptans, thyroid hormones, parasympathomimetics, sympathomimetics, antiepileptics, pharmaceutical combination preparations, chloride channel activators, antidepressants, neuroleptics, benzodiazepines, sedatives, antipsychotics, glucocorticoids, antirheumatic drugs, were grouped together (specified information is available from the authors). A total of 89 participants (32.6%) reported taking at least one medication with potential effect on the cardiovascular system and/or HPA axis, including 83 patients and 6 healthy controls.

### Vividness of imagery check

At the end of the procedure, all participants had to rate the imagination task on a scale from 0 (little vivacious) to 10 (very vivacious) to capture the participant’s ratings of the perceived vivacity of the imagination.

### Implicit memory check

To detect the level of attention and confrontation with the material an implicit picture recognition task was presented to the participants showing 10 images taken from the magazine of the corresponding condition and 10 pictures from another issue of the magazine. Participants had to decide whether each picture was in the magazine or not and performance was evaluated according to the proportion of correct answers.

### Statistical data analysis

Data were analysed with IBM SPSS Statistics for Macintosh, Version 24. Questionnaire data for two participants were missing and were therefore removed. Analyses were performed on log-transformed HRV and log-transformed salivary cortisol data.

ERD were operationalized by using the DERS total score as predictor in our statistical models without any categorization. ERD were highly confounded with mental disorder categories (F_3,269_ = 106.5, p < .001, h^2^ = .54 based on analysis of variance for differences in ERD among groups). Consequently, and because the impact of diagnostic subgroups was not in the primary focus of this study, these were not considered in any analyses. For the same reasons we did not attempt to analyze the impact of ERD within diagnostic groups (moderator analysis) as this would have led to potentially very small variances of DERS scores.

To test whether participants with high ERD showed lower HF-HRV and higher salivary cortisol at baseline, general linear models were calculated with DERS and condition (experimental vs. control) as predictors. We further conducted a linear mixed model to test whether changes in both HF-HRV and salivary cortisol during time intervals baseline, vivid imagination, and recovery depended on participants’ DERS values and whether this relationship varied with experimental condition. Thus, this model contained measurement time points (baseline, vivid imagination and recovery) as within-subjects predictor and condition (experimental or control) and the DERS as between-subjects predictors, including all interactions. The same linear mixed model was used for the outcome self-reported mood variable to test whether changes in mood after vivid imagination depended on participants’ DERS values and whether this relationship varied with experimental condition.

Apart from BMI and age, we also included implicit memory check, vividness of imagery check and use of hormonal contraceptives and medication as covariates in order to control for effects of the moderate stress induction. *p*-values were corrected for multiple testing within each of the three models using Benjamini and Hochberg’s method [52]. The number of *p*-values was thereby 4 for testing HF-HRV and salivary cortisol at baseline, 9 for testing changes in mood, HF-HRV and salivary cortisol between baseline and vivid imagination and 6 for testing changes in HF-HRV and salivary cortisol between vivid imagination and recovery. For transparency reasons, both uncorrected and corrected *p*-values are reported in this order, but only the corrected ones are referred to when discussing the results.

## Results

### Sample characteristics

The 273 participants (172 patients and 101 healthy controls) were randomly assigned to EC and CC. Table 1 displays characteristics of the entire sample and, for clarity and only in this table, by subsamples low or high ERD (according to median split of the DERS). The low DERS group consisted of 68.6% healthy controls and 31.4% patients. The high DERS group of 3.8% healthy controls and 96.2% patients.

**Table 1:** Sample characteristics.

	All	Low ERD DERS score = 39–96	High ERD DERS score = 97–171	*F*
*n*	273	140	133	
DERS	96.71 (31.32)	70.56 (15.98)	124.23 (16.25)	756.707***
Age	22.87 (3.94)	22.33 (3.37)	23.50 (4.41)	6.003*
BMI	21.75 (4.48)	21.59 (3.12)	21.94 (5.57)	.392
**Mood response (mean/ *SD*) (range 0–100)**				
*n*	273	140	133	
Before VI	67.49 (19.71)	78.35 (14.14)	56.05 (18.25)	126.367***
*n*	272	139	133	
After VI	65.99 (22.32)	77.55 (16.14)	53.91 (21.50)	104.496***
**HPA response (mean/ *SD*): Salivary cortisol (nmol/l)**				
*n*	272	140	132	
**Baseline**	5.78 (4.16)	5.58 (3.57)	6.00 (4.71)	.720
*n*	271	138	133	
**Vivid imagination**	5.13 (3.64)	4.89 (3.19)	5.37 (4.06)	1.167
*n*	270	138	132	
**Recovery**	5.37 (4.3)	5.16 (3.90)	5.59 (4.69)	.672
**ANS response (mean/ *SD*)**				
**Baseline**				
*n*	241	119	122	
HR (bpm)	70.54 (11.09)	69.14 (9.69)	71.91(12.18)	3.793
meanRR	881.85(149.41)	897.30(138.51)	866.79(158.44)	2.528
HF-HRV ln(ms^2^)	6.81 (1.12)	7.12 (.97)	6.50 (1.18)	19.612***
LF-HRV ln(ms^2^)	7.15 (.80)	7.40 (.69)	6.90 (.82)	26.999***
LF/HF ratio	1.87 (1.62)	1.68 (1.27)	2.04 (1.89)	3.032
TPHR ln(ms^2^)	7.86 (.84)	8.12 (.72)	7.60 (.87)	25.464***
**Vivid imagination**				
*n*	239	119	120	
HR (bpm)	69.48 (11.15)	67.64 (10.01)	71.30 (11.94)	6.612*
Mean RR	894.24 (151.70)	916.88 (147.04)	871.79 (153.51)	5.374*
HF-HRV ln(ms^2^)	6.65 (1.19)	7.04 (1.04)	6.26 (1.21)	28.401***
LF-HRV ln(ms^2^)	6.82 (.83)	7.02 (.76)	6.62 (.86)	14.543***
LF/HF ratio	1.70 (1.53)	1.41 (1.26)	2.00 (1.70)	9.367**
TPHR ln(ms^2^)	7.62 (.89)	7.90 (.78)	7.34 (.91)	25.751***
**Recovery**				
*n*	237	117	120	
HR (bpm)	69.88 (11.08)	68.35(9.84)	71.36(12.02)	4.430*
Mean RR	888.1214 (150.05)	905.94 (144.24)	870.75 (154.14)	3.291
HF-HRV ln(ms^2^)	6.8 (1.17)	7.06 (1.04)	6.54 (1.24)	12.345**;
LF-HRV ln(ms^2^)	7.03 (.93)	7.06 (1.04)	6.54 (1.24)	19.613***
LF/HF ratio	1.90 (1.90)	1.95 (2.08)	1.83 (1.72)	.224
TPHR ln(ms^2^)	7.80 (.93)	8.06 (.77)	7.54 (1.00)	20.465***

Age in years; BMI = Body Mass Index (kg/m2); DERS = Difficulties in Emotion Regulation Scale; mean RR = RR interval; TPHR = total power of all spectrum; VI = Vivid imagination. Assumption of homogeneity was violated for the variables BMI and age, Mood, Baseline LF-HRV, LF/HF ratio and TPHR, vivid imagination LF/HF ratio and TPHR, recovery HF-HRV, LF-HRV and TPHR therefore sig. are reported using Brown-Forsythe test.

Notes. Sample sizes varied among the different variables due to missing values.

*p<0.05

** *p*<0.01

*** *p*<0.001

### Effects on self-reported mood

**Fig 2:**
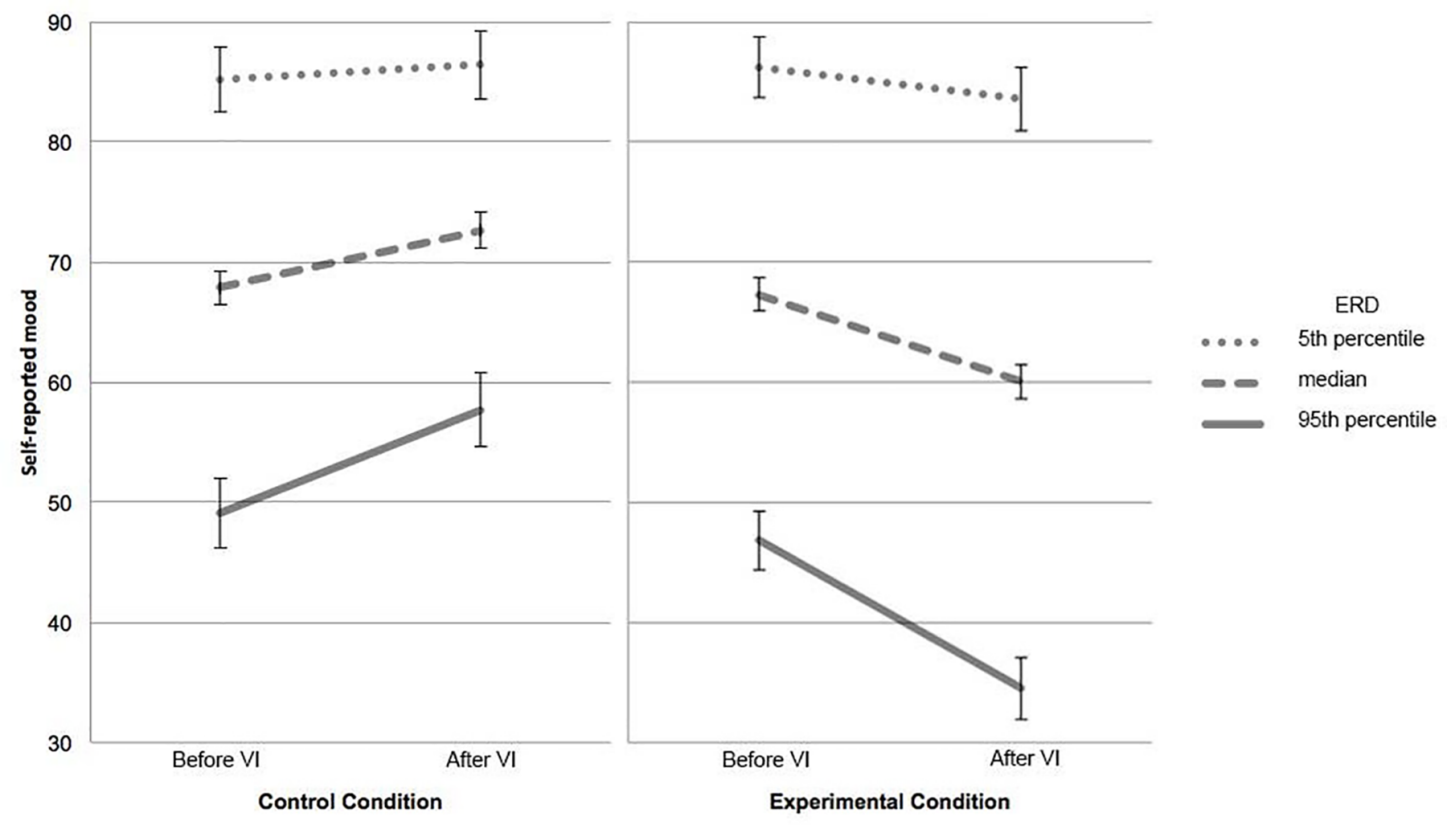
Mood across conditions and levels of DERS over the course of vivid imagination. VI = Vivid imagination. Lines denote predicted values for mixed model including standard errors. To meaningfully cover the range of ERD values encountered in the sample, trajectories of the median, 5^th^ and 95^th^ percentiles of the DERS are shown.

Linear mixed models revealed a significant time x condition interaction (*F*(1,247) = 67.518, *p* < .001/ < .001), indicating an overall increase in self-reported mood in the CC and a decrease in the EC after vivid imagination as expected. There was no significant time x DERS interaction (*F*(1,247) = 1.494, *p* = .223/.287). Furthermore, there was a significant three-way interaction of time x condition x DERS (*F*(1,247) = 13.202, *p* < .001/ < .001). Thus, during vivid imagination, participants with higher DERS values showed a stronger decline in self-reported mood compared to participants with lower DERS values in the thin ideal condition but a stronger increase with increasing DERS values in the CC (Fig 2).

### Results for HRV

Analyses showed neither a main effect of DERS on baseline HF-HRV (*F*(1,219) = 1.240, *p* = .267/.809), nor an interaction of condition x DERS (*F*(1,219) = .059, *p* = .809/.809). Further there was no effect of DERS on HRV between baseline and vivid imagination such as a three-way interaction between time, condition and DERS (*F*(1,217) = .043, *p* = .837/.837) or an interaction of time x condition (*F*(1,217) = 3.868, *p* = .050/.113), or for time x DERS (F(1,217) = 5.191, *p* = .024/.072). In addition, there was no effect on HRV between vivid imagination and recovery including three-way interaction between time, condition, and DERS (*F*(1,214) = 2.589, *p* = .109/.164), nor a two-way interaction time x condition (*F*(1,215) = 3.510,*p* = .062/.124), or for time point x DERS (*F*(1,214) = 4.412, *p* = .037/.111).

### Results for salivary cortisol

Analyses showed neither a main effect of DERS on baseline salivary cortisol levels (*F*(1,246) = .455, *p* = .501/.809), nor an interaction of condition x DERS (*F*(1,246) = .111, *p* = .739/.809). Similar, there was no effect on salivary cortisol between baseline and vivid imagination including a three-way interaction between DERS, time and condition (*F*(1,245) = 1.923, *p* = .167/.263) or any two-way interactions of time x condition (*F*(1,245) = .734, *p* = .392/.441) and of time x DERS (*F*(1,245) = 1.853, *p* = .175/.263). Finally, there was no effect of DERS on salivary cortisol between vivid imagination and recovery including a three-way interaction between DERS, time and condition (*F*(1,245) = 5.542, *p* = .019/.111) and no two-way interactions of time x condition (*F*(1,245) = .015, *p* = .902/.902) and of time x DERS (DERS, *F*(1,245) = .880, *p* = .349/.419).

## Discussion

The aim of the present study was to examine whether changes occurring in mood, HRV and salivary cortisol following a moderate daily stress induction (laboratory thin ideal exposure and vivid imagination) are associated with different range of ERD in a large sample consisting of healthy and mentally ill young women.

We hypothesized that higher self-reported trait ERD are linked to a higher decrease in self-reported mood after vivid imagination of thin ideals. Higher trait ERD should be observed in a lower HF-HRV at rest and higher baseline cortisol levels, as well as in smaller HRV changes during vivid imagination of thin ideals but reduced cortisol responses and slower recovery of salivary cortisol levels after vivid imagination of thin ideals compared to landscapes.

In line with our previous research [33], vivid imagination of the thin ideal had a negative impact on self-reported mood in young women. Further, results showed that there was an increase of positive mood in the CC, whereas a stronger increase of negative mood was revealed in the EC with increasing ERD. It is reasonable to assume that exposure to thin ideals and vividly imagining them, impose a negative affect. Especially for participants who tend to engage in dysfunctional emotional processing [53]. However, in our sample the imagination of landscapes might have led to a shift of attention towards more positive stimuli, especially in those participants characterized by high ERD. This is an interesting finding as it illustrates that engaging in vivid imagination of non-idiosyncratic problem-related topics, such as pictures of landscapes, might intervene with ruminative processes [38], as demonstrated by the subsequent increase in mood.

Our results corroborate the role of ERD regarding the psychological stress response to vivid imagination of the thin ideal. A decrease in mood after vivid imagination was observed in a similar study by Wyssen et al. [33] among healthy women, where results depended on eating disorder symptomatology. In fact, the decrease in mood in the experimental condition could only be found in subjects with more pronounced eating disorder symptomatology. Our results should therefore be interpreted with caution. Although ERD contribute to the better understanding of the effect of vivid imagination on mood, the effect of other factors related to psychopathology or to the specificity of the stressor cannot be ruled out.

Regarding biological measurements, trait ERD were not linked to baseline HF-HRV. Further, our assumption that low HF-HRV is linked to low emotion regulation abilities in challenging situations was not confirmed. These findings are partly in line with the results of Berna et al. [19], who did not find differences in smaller study samples with low ERD (*n* = 18) and high ERD (*n* = 19) concerning HF-HRV at rest. However, in their study high ERD showed a slower recovery after stress than low ERD. In contrast to our study, Berna et al. [19] did not control for medication or use of contraceptives although they excluded subjects with substance use disorder from their sample. In our study, alcohol or drug consumption was not an exclusion criterion in order to preserve clinical reality of the sample. The comparability of these studies is therefore limited, due to varying sample size and characteristics.

Our assumption that ERD were related to higher baseline salivary cortisol levels was also not confirmed. Further, our results showed a general decline of salivary cortisol independently of ERD and condition between baseline and recovery. As outlined by Gilbert et al. [35], there is a lack of studies assessing the impact of ERD on the HPA axis and comparable findings are therefore scarce.

To sum up, the hypothesis that thin ideal exposure and vivid imagination would trigger a biological stress response was not confirmed, even though results on self-reported mood underline the negative impact of our stress induction paradigm [29,30]. This lack of a biological stress response to a daily stressor applied in the laboratory is noteworthy considering that our study fulfilled several of the suggestions of Campbell and Ehlert [54]. That is to increase the validity of results by focusing on a dynamic pattern of mood change, using precise time and context records, including large samples and considering the effect of mental illness or related biological factors such as menstrual cycle or medication. Our findings of discordant self-report and biological stress response patterns are in line with existing literature which generally showed that stress inducing paradigms using thin ideal or body confrontation have failed to reveal consistent results in ANS or HPA axis activation [27,54]. Even following mirror confrontation, where pronounced negative emotions and cognitions were reported in patients with eating disorders, both HRV and salivary cortisol remained stable [27]. A similar dissonance between self-report and physiological response has also been reported in AN patients [55].

Additionally, the discrepancy between the influence of emotion regulation mechanisms on the psychological level and the absence of physiological arousal may be due to the direct impact of varying state-related emotion regulation strategies not captured by the DERS. Another line of argument assumes, as in Gross and Levenson [53], that physiological activation depends on whether a stimulus sufficiently triggers emotional expression or not. This assumption has been confirmed by Mauss et al. [56], who showed that the type and intensity of a stimulus determine the degree of concordance between psychological and physiological outcomes. According to the authors, specific emotions with a cognitive component such as anxiety may lead to a more pronounced imbalance between the extent of the psychological and the physiological reaction compared to other emotions such as fear. Furthermore, the imbalance is related to the intensity of elicitation of a target emotion, as physiological response may have a higher activation threshold. We therefore think that future studies should investigate in more detail the immediate effect of thin ideal exposure and vivid imagery on cognitions and affect. The effects of frequency and intensity of exposure of a moderate daily stressor on biological stress responses also merits further study.

Several limitations must be considered in our study. First, the complexity of the biological processes involved may be insufficiently captured by the specific measurement time points in our design. This has been recently demonstrated by Goodman et al. [57] for salivary cortisol. Additionally, it has to be mentioned, that the length of periods to assess HF-HRV differed from baseline compared to during stress exposure and thereafter. In general, spectral values should be less affected by time length than time domain values, but this potential limitation has to be kept in mind. As previously shown in Wyssen et al. [33], a general anticipatory arousal to the experiment and a subsequent habituation to the experimental manipulation along with the combined effects of media exposure/vivid imagination, is to be expected. Furthermore, self-reported intensity of vividness of imagery was assessed, but additional analyses would be necessary to define the valence of its core content. Second, our design did not involve the assessment of state-related emotion regulation while being exposed to thin-ideals or while vividly imagining them compared to the CC. Instead, we examined, whether and how self-reported trait-like ERD influenced stress responses to a laboratory stress task. In other words, the design of our study does not allow drawing conclusions about how state-related emotion regulation influences the response when facing a stressor. We also did not assess the impact of the type of emotion experienced during thin ideal imagination. Even though it is known that, certain physiological responses are associated to specific emotions [18]. In addition, we cannot rule out the effect of other psychopathological features or attentional processes during the exposure with our specific stressor. In sum, our findings point to the role of ERD, but also accentuate that ERD does not fully explain the impact of vivid imagination of the thin ideal on mood. Third, the healthy study sample was solely recruited in Switzerland, while the clinical sample included participants of both countries, which might limit the comparability of the findings. Nor did the study include male participants or a broader age range, although age may influence the impact of ERD on the evolution of different pathologies.

Apart from these limitations, the large sample size and the inclusion of mentally healthy and participants suffering from a range of mental disorders add to the strength of the study. Furthermore, the study contributes to the exploration of physiological processes during a moderate daily stressor exposure.

In sum, this study showed a clear discrepancy between psychological and physiological responses as previously detailed out by Campbell and Ehlert [54]. The discrepancy between psychological and biological measures of stress responses is a well-known phenomenon in anxiety patients during treatment and especially in phobic patients showing heightened cortisol responses in height exposure despite improved subjective and behavioral responses [58]. We applied a moderate daily stressor with salience for our young female population in the laboratory and found a subsequent deterioration in mood, relative to the CC, where an increase in mood was observed, especially in women with high ERD. This is an important finding, as first, there might be additive negative psychological and biological effects of repeated exposure and related information processing in a longitudinal perspective. Second, enduring ERD are known to be associated with impaired inhibition of irrelevant emotional information, less goal-oriented behavior and impaired ability to reappraise stressful situations [59]. These mechanisms are per se relevant as they predict mood, anxiety, and EDs [4,59]. Added knowledge on the relation between ERD and biological correlates of stress response is of high interest, as impaired stress reactivity is associated with impaired mental health and increased symptom severity in a broad range of mental disorders [60–63]. If future studies confirm that ERD relate to impaired stress responses to challenges, emotion regulation trainings could be implemented in either prevention or treatment trials; similar to what has already been shown to be effective for depressive and anxiety disorders [64,65].

Our findings highlight the necessity to investigate ecological valid moderate stressors, occurring repeatedly on various levels of intensity, in order to investigate the repercussions of daily stressors on psychological and physiological levels.
